# Phylogenetic ancestry of Metamonada proteins points to a common origin of mitochondria in all eukaryotes

**DOI:** 10.1093/molbev/msag175

**Published:** 2026-07-17

**Authors:** Kristína Záhonová, Pavel Doležal, Jan Tachezy, Julius Lukeš, Dave Speijer, Vladimír Hampl

**Affiliations:** Department of Parasitology, Faculty of Science, Charles University, BIOCEV, Vestec, Czechia; Institute of Parasitology, Biology Centre, Czech Academy of Sciences, České Budějovice, Czechia; Life Science Research Centre, Faculty of Science, University of Ostrava, Ostrava, Czechia; Division of Infectious Diseases, Department of Medicine, University of Alberta, Edmonton, Alberta, Canada; Department of Parasitology, Faculty of Science, Charles University, BIOCEV, Vestec, Czechia; Department of Parasitology, Faculty of Science, Charles University, BIOCEV, Vestec, Czechia; Institute of Parasitology, Biology Centre, Czech Academy of Sciences, České Budějovice, Czechia; Faculty of Sciences, University of South Bohemia, České Budějovice, Czechia; Amsterdam UMC, AMC, Department of Medical Biochemistry, Amsterdam, The Netherlands; Department of Parasitology, Faculty of Science, Charles University, BIOCEV, Vestec, Czechia

**Keywords:** Metamonada, Alphaproteobacteria, mitochondria, MROs, LECA, endosymbiosis

## Abstract

Hypotheses concerning eukaryogenesis, the evolution of eukaryotic cells, differ in the relative timing of mitochondrial acquisition. Recently, a serial endosymbiotic hypothesis proposed that hydrogenosomes and mitosomes (MROs) in Metamonada originated from an independent endosymbiosis, later replaced by Alphaproteobacteria-related mitochondria, contradicting the paradigm of mitochondrial presence in the last eukaryotic common ancestor. This serial endosymbiotic hypothesis implicitly predicts the scarcity of alphaproteobacterial genes from Metamonada genomes, because they never contained this endosymbiont. We tested this prediction using a set of 1,399 and 97 proteins inferred for the Metamonada ancestor and confined to their MROs, respectively. We detected five and 14 orthologous groups (OGs) with alphaproteobacterial affiliation in the respective datasets. None of these OGs was present in oxymonads, a Metamonada subgroup lacking MROs, thus serving as blank references. Our data are therefore consistent with the ruling paradigm that mitochondria and MROs originated from an Alphaproteobacterium during a single common endosymbiosis.

## Introduction

The evolution of eukaryotic cells was shaped by multiple symbiogenic events, giving rise to semiautonomous organelles such as mitochondria, plastids, chromatophores, nitroplasts, spheroid bodies, and, possibly, others (Margulis 1981; Kneip et al. 2008; Nowack et al. 2008; Archibald 2015; Sibbald and Archibald 2020; Coale et al. 2024). Consequently, eukaryotic genomes are chimeric, with genes derived from the archaeal “host” inherited vertically and from bacterial ancestors of the above-mentioned organelles inherited via endosymbiotic gene transfer (EGT) (Esser et al. 2004; Pisani et al. 2007; Tobiasson et al. 2026). A surprising number of additional genes originated from other prokaryotes without endosymbiosis through horizontal gene transfers (HGTs) (Koonin 2010a; Speijer 2015; Sibbald et al. 2020; Tobiasson et al. 2026). This extremely chimeric nature of the genome of the last eukaryotic common ancestor (LECA) has given rise to several models of eukaryogenesis, differing in the prokaryotic lineages involved and the relative timing of entry (Martin and Muller 1998; Moreira and Lopez-Garcia 1998; Pittis and Gabaldón 2016; Speijer 2017; Imachi et al. 2020; Krupovic et al. 2020). However, none of these models questions the alphaproteobacterial origin of mitochondria. Asgard archaea, the closest known eukaryotic relatives (Eme et al. 2023; Zhang et al. 2025), contributed massively to the eukaryotic genomes, as approximately 50% of eukaryotic genes are affiliated with this group (Tobiasson et al. 2026). Of the bacteria-related genes, only a small minority (4%–7%) is affiliated specifically with Alphaproteobacteria, the mitochondrial predecessors (Koonin 2010b; Rochette et al. 2014; Ku et al. 2015; Vosseberg et al. 2020; Santana-Molina et al. 2025; Tobiasson et al. 2026). This “underrepresentation” has led to speculation regarding the importance and uniqueness of the endosymbiosis leading to mitochondria (Al Jewari and Baldauf 2023).

The Metamonada constitute a deep-branching eukaryotic group of great evolutionary interest. They are subdivided into five lineages (Parabasalia, Anaeramoebae, Fornicata, barthelonids-skoliomonads [BaSk], and Preaxostyla) (Stairs et al. 2021; Williams et al. 2024). Endobiotic trichomonads and diplomonads from the parabasalid and fornicate groups, respectively, were previously considered amitochondriate “archezoans,” a hypothetical group branching off the eukaryotic stem prior to the acquisition of mitochondria (Cavalier-Smith 1993; Cavalier-Smith and Chao 1996). However, these protists are now known to contain highly reduced mitochondrion-related organelles (MROs), such as hydrogenosomes (Lindmark and Müller 1973; Hrdy et al. 2004; Jerlström-Hultqvist et al. 2013) and mitosomes (Tovar et al. 2003). More recently, endobiotic metamonads of the preaxostylan Oxymonadida group were shown to have completely lost all traces of the organelle itself (Karnkowska et al. 2016; Novák et al. 2023).

Following a debate over the evolutionary position of anaerobic eukaryotes, the current consensus is that the LECA contained a facultatively aerobic mitochondrion (reviewed in [Roger et al. 2017; Hampl and Roger 2024]), which was stepwise altered and, in some lineages, extensively reduced. This came about as a result of the interplay of HGT furnishing non-mitochondrial alternatives, such as a MIS (Fe-S) assembly system or FeFe-hydrogenases (Lewis et al. 2020; Záhonová et al. 2023), and colonizing niches low in molecular oxygen (Yarlett and Hackstein 2005; Müller et al. 2012; Tachezy 2019). Such a functional reduction is nicely illustrated by microsporidia (Williams et al. 2002; Haag et al. 2014) or Archamoebae, such as *Entamoeba histolytica* (Mai et al. 1999; Tovar et al. 1999; Záhonová et al. 2023). Because microsporidians and *Entamoeba* occupy distinct phylogenetic positions among aerobic lineages in the eukaryotic tree, the identification of these MROs, and those of other anaerobic taxa, as *bona fide* reduced mitochondria is no longer questioned (Kang et al. 2017; Burki et al. 2020; Galindo et al. 2022; Speijer 2023). Yet, while these examples firmly establish that anaerobiosis in eukaryotes arose through secondary reduction, the situation in metamonads presents a more complex and debated case. All current known metamonads are heterotrophic, anaerobic protists. Since they constitute a deep-branching lineage, their exact position remains disputed (Burki et al. 2020; Al Jewari and Baldauf 2023; Williamson et al. 2025). Thus, in a recent phylogenetic reconstruction, the position of Metamonada (illustrated by *Anaeramoeba*) was poorly resolved and the metamonad placement at the root of the eukaryotic tree (or elsewhere) could not be robustly excluded (Williamson et al. 2025).

Recently, it was proposed that metamonad MROs are *not* derived from the alphaproteobacterial ancestor of mitochondria, but from a prior uptake of an anaerobic bacterium (Al Jewari and Baldauf 2023). To assess the robustness of this hypothesis, we phylogenetically dissected the alphaproteobacterial contribution to the ancestral and MRO proteins of Metamonada. We provide strong evidence that the common ancestor of this group possessed an organelle of alphaproteobacterial ancestry, i.e. a mitochondrion.

## Results

### A small fraction of ancestral metamonad proteins is affiliated with Alphaproteobacteria

**Figure 1:**
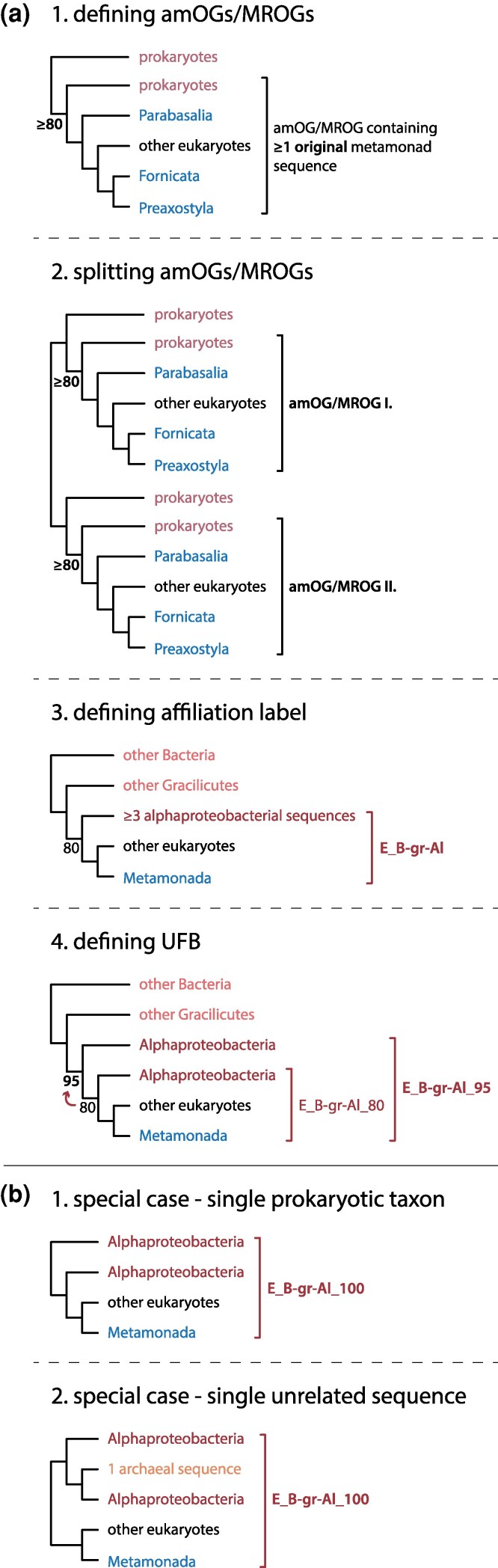
Assessment of the prokaryotic affiliation of amOGs/MROGs. a) Assessment was performed in four steps. 1. The amOG was defined as the smallest subtree from the unrooted gene tree that received UFB support ≥80 using the following criteria: (i) presence of at least one metamonad from the original amOGs in Table S1 and (ii) presence of representatives from both sides of the deepest split of Metamonada (i.e. Parabasalia/Anaeramoebae and Fornicata/BaSk/Preaxostyla). The MROG was defined similarly by the presence of at least one *bona fide* MRO sequence, but criterion (ii) was not required. Both amOGs and MROGs were allowed to include branches of other eukaryotes or prokaryotes. 2. If more than one such amOG/MROG was present in the tree, the following steps were performed separately for each of them, and all were included in the statistics. 3. For each amOG/MROG, the affiliation label was defined as follows. If eukaryotes in the amOG/MROG were represented only by metamonads, the affiliation label started with “M”. If other eukaryotes were present, the label started with “E.” The label continued with the prokaryotic affiliation, determined as the lowest taxonomic rank common for the three closest prokaryotes (forming one or more branches) using the ranking and abbreviation scheme in column F of Table S2. As an example, E_B-gr-Al designates an OG containing Metamonada with other eukaryotes and at least three representatives of Alphaproteobacteria (column C in Table S2). 4. The last element of the affiliation label was the UFB support. If moving up the backbone of the tree did not change the affiliation label but increased the UFB support, the highest UFB support was accepted. b) Two additional rules were applied in special cases. 1. If only metamonads/eukaryotes and one prokaryotic clade were present in the whole tree, the UFB support was considered to be 100. 2. If a single unrelated sequence broke a taxonomically homogeneous amOG/MROG, e.g. one archaeal sequence in amOG/MROG containing only eukaryotes and Alphaproteobacteria, the sequence was ignored.

To evaluate the contribution of prokaryotic lineages to Metamonada genomes while avoiding more recent lineage-specific HGTs, we focused on 1,399 orthologous groups (OGs) of putative ancestral metamonad proteins (Table S1). Each OG was first enriched for homologs from a custom-built database (Table S2) containing all available metamonad datasets and representation of Eukaryota, Bacteria, and Archaea. To improve sensitivity to genes originating from Alphaproteobacteria, this database was intentionally enriched with phylogenetically diverse datasets of this bacterial subgroup. After filtering, 965 OGs were retained and subjected to phylogenetic inference (Table S3a). The inspection of the trees identified 954 clades (ultrafast bootstrap [UFB] support ≥80) containing a broad representation of metamonads, as well as other eukaryotes and prokaryotes. We call these clades amOGs, and the prokaryotic affiliation of each amOG was determined based on the taxonomy of the contained prokaryotes (Fig. 1).

**Figure 2:**
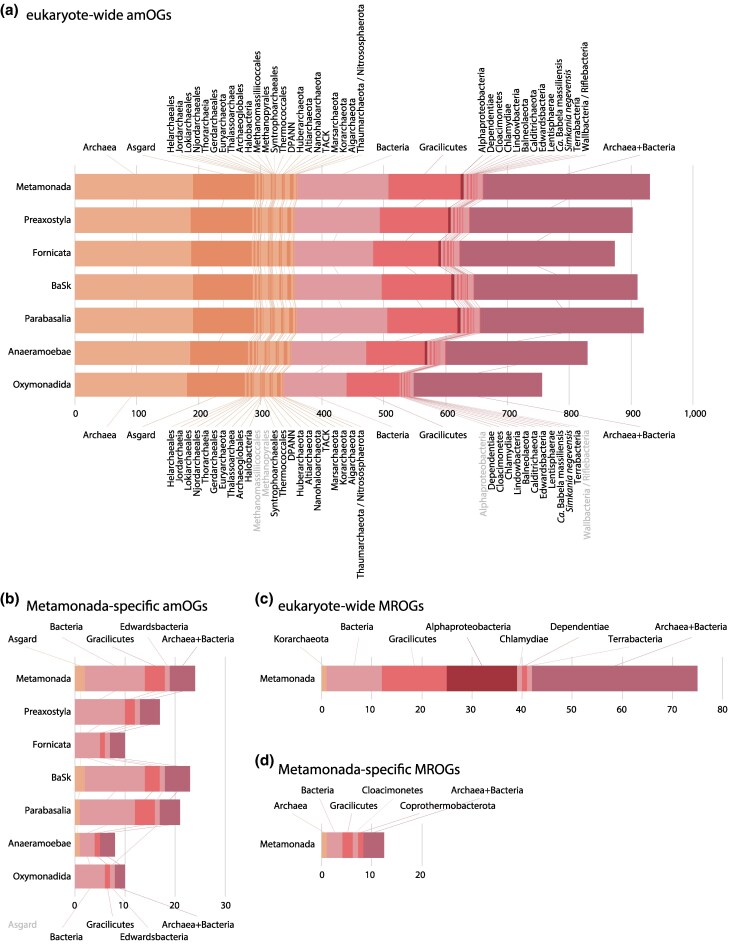
Counts of eukaryote-wide (a, c) and Metamonada-specific (b, d) amOGs and MROGs. Phylogenetic affiliation of amOGs and MROGs to particular groups of prokaryotes is shown as bar charts. Names of prokaryotic groups not affiliated with any Oxymonadida protein are in gray in the bottom labels. For further details, see Tables S3 and S7.

The vast majority (930) of amOGs also contained genes from other eukaryotes besides Metamonada (Table S3b), and thus likely represent genes vertically inherited by Metamonada from deeper eukaryotic ancestors, including the LECA. Out of these eukaryote-wide amOGs, 360 (38.7%) were robustly affiliated to Archaea, 301 (32.4%) to Bacteria, while the affiliation of the remaining 269 (28.9%) could not be unambiguously established because sequences from both prokaryotic domains were present, and thus were assigned as “Archaea+Bacteria” (Fig. 2a). From the archaeal amOGs, 192 (53.3%) could not be further assigned to any specific archaeal subgroup, however, 114 (31.7%) were assigned to Asgard, and the remaining 54 (15%) to archaeal subgroups other than Asgard. Almost half of the bacterial amOGs (148, 49.2%) could not be further assigned to any specific bacterial subgroup. The second largest group was constituted by amOGs affiliated with Gracilicutes (117, 38.9%), a supergroup containing Alphaproteobacteria. Only five amOGs (0.5%) were specifically related to Alphaproteobacteria (Figure S1). These represented proteins involved in protein import, processing, and folding (Pam18/q2005667, Hsp70/q2000295, and MPP/q2004851) and amino acid metabolism (GCS-P/q2004773 and ornithine decarboxylase/q2001279). To reveal lineage-specific affiliation profiles, subsets of 930 amOGs, containing representatives of individual Metamonada lineages, were extracted (Table S3c) and compared. The differences were small; however, the preaxostylan subgroup Oxymonadida showed a lower proportion of Bacteria-affiliated amOGs (211, 27.9%) and none specifically affiliated with Alphaproteobacteria (Fig. 2a).

Only 24 amOGs contained no eukaryotes besides Metamonada (Table S3b), thus likely representing HGTs to the Metamonada common ancestor. The majority of these (17, 70.8%) was affiliated with Bacteria, and, again, the affiliation profile was similar among the lineages (Fig. 2b). Proteins encoded by these genes mostly act as metabolic enzymes and membrane transporters (Table S4).

### MRO proteomes are enriched for proteins affiliated with Alphaproteobacteria

Our next analyses were focused on a subset of metamonad proteins with a putative MRO function. Using published MRO proteomes of *Pentatrichomonas hominis*, *Paratrimastix pyriformis*, and *Spironucleus salmonicida*, as well as in-house compiled and curated MRO proteomes of *Trichomonas vaginalis* (Table S5) and *Giardia intestinalis* (Table S6), and confidently predicted MRO proteins (Füssy et al. 2021; Stairs et al. 2021; Novák et al. 2023; Makki et al. 2024; Williams et al. 2024), we compiled a set of 97 *bona fide* MRO-derived proteins. Phylogenetic inferences revealed 87 clades (UFB support ≥80) containing representation of Metamonada species, as well as other eukaryotes and prokaryotes. We call these clades MROGs, and the prokaryotic affiliation of each MROG was determined based on the taxonomy of the contained prokaryotes (Fig. 1). 75 MROGs also contained other eukaryotes, while 12 were Metamonada-specific (Fig. 2c and d; Table S7). For both groups, the affiliation profile differed markedly from that of amOGs. Archaeal affiliations were negligible (only a single case in each dataset), whereas bacterial affiliations predominated (41, 54.7% and 7, 58.3%, respectively). Among these, 14 MROGs (16.1%) showed alphaproteobacterial affiliation (Figure S2). These represent proteins involved in protein import, processing, and folding (Pam16, Tim44, MPP, Hsp70, and Cpn60), Fe-S cluster assembly (IscS, IscA, and frataxin), and amino acid (GCS-P1, -P2, -L, and -T) and energy metabolism (NuoF and SCSa).

## Discussion

In this study, we sought to assess the alphaproteobacterial contribution to the ancestral genomes of Metamonada, a deep-branching group of protists lacking classical mitochondria, which, in the recent reincarnation of the Archezoa hypothesis (Al Jewari and Baldauf 2023), were proposed to descend from eukaryotes prior to mitochondrial endosymbiosis (Cavalier-Smith 1993). Our data suggest a roughly equal contribution of the archaeal and bacterial domains to the Metamonada gene repertoire, which conflicts with several earlier studies assessing the relative contributions in diverse eukaryotes, reporting a higher proportion of genes related to Bacteria (Esser et al. 2004; Pisani et al. 2007). However, it agrees with a recent extensive study (Tobiasson et al. 2026), and this agreement clearly corresponds with the inclusion of the Asgard archaeal datasets in these two latest analyses. A notable difference from the results based on the general eukaryotic dataset (Tobiasson et al. 2026) is a lower number of OGs affiliated with Alphaproteobacteria, namely ∼6% in the eukaryotic (Tobiasson et al. 2026) vs. 0.5% in the Metamonada dataset (amOGs). Yet, proteins affiliated with Alphaproteobacteria are highly enriched (16%) among those associated with MROs (MROGs). Notably, no Alphaproteobacteria-derived genes were identified using our approach in oxymonads, a group lacking both mitochondria and MROs, which may be regarded as a blank reference dataset.

Identification of genes affiliated with Alphaproteobacteria in Metamonada and their enrichment in MRO proteomes conflict with the hypothesis of Al Jewari and Baldauf (2023). These genes encode proteins functioning in essential systems involved in biogenesis of mitochondria (mitochondrial import and folding, FeS cluster assembly, amino acid metabolism), which indicate that they have not originated from a set of random HGTs but accompanied the emergence of these organelles. Notably, the vast majority of Alphaproteobacteria-affiliated OGs recovered robustly supported clades containing Metamonada with other eukaryotes branching sister to/nested in Alphaproteobacteria (Figs. S1 and S2). The simplest explanation for this pattern is that homologs in Metamonada and other eukaryotes originated from the same prokaryote, an endosymbiont that gave rise to both mitochondria and MROs. This is in line with classical studies of heat shock proteins and FeS cluster assembly enzymes in Metamonada, which all exhibit clear mitochondrial (alphaproteobacterial) affiliations (Bui et al. 1996; Horner et al. 1996; Roger et al. 1996, 1998; Tachezy et al. 2001; Hampl et al. 2008; Novák et al. 2023), and aligns with the current paradigm of a common origin of mitochondria and MROs in all extant eukaryotes (Roger et al. 2017; Bravo-Arévalo 2025; Speijer 2025). The markedly lower number of genes affiliated with Alphaproteobacteria in metamonads can be explained by a transformation of canonical mitochondria to MROs, accompanied by a reduction of the organellar proteome and an increased sequence divergence of the MRO proteins.

In the same vein, the complete loss of MROs in oxymonads corresponds with the drop in the number of Alphaproteobacteria-affiliated genes below the level at which we can detect them. Oxymonads are uniquely suited to observe the fate of genes derived from an endosymbiont after its physical remnant—the organelle—vanished. A widely accepted view holds that the protein products of some EGT-acquired genes perform functions outside the organelle (Martin et al. 2002; Bolte et al. 2015), and thus remain unaffected by its loss (Bock and Timmis 2008). However, this category of genes may be very small, if any. Thus, all genetic traces may vanish with the organelle (Keeling 2024), as illustrated by our data from oxymonads.

**Figure 3:**
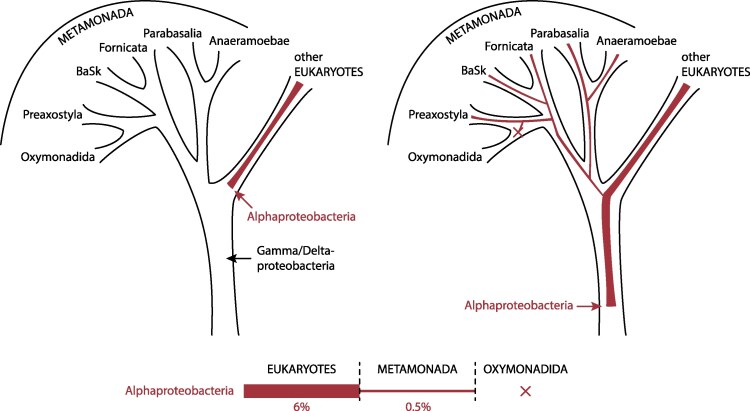
Alternative hypotheses of mitochondrial evolution and their fit to the data observed. The serial endosymbiotic (Al Jewari and Baldauf 2023) and “mitochondrion-in-LECA” (Roger et al. 2017) hypotheses differ in their predictions about the presence of genes affiliated with the Alphaproteobacteria (indicated by red lines inside the trees) in eukaryotic genomes. The serial endosymbiotic hypothesis (left) predicts these genes to be scarce in Metamonada, since they diverged from the main trunk before the mitochondrial endosymbiosis. The mitochondrion-in-LECA hypothesis (right) assumes these genes are present in all eukaryotes. Observed data (bottom) are consistent with the mitochondrion-in-LECA hypothesis, while the significant reduction of these genes in Metamonada can be explained by remodeling of their mitochondria to MROs upon assuming an anaerobic lifestyle. The absence of these genes in oxymonads correlates with the complete loss of the organelle. The percentages of genes affiliated with Alphaproteobacteria were taken fromTobiasson et al. (2026) (core dataset) for eukaryotes and estimated for Metamonada in this study.

In conclusion, we provide evidence from an extensive analysis of currently available genomic data supporting the reigning paradigm (Fig. 3), namely that *all* extant eukaryotes, including Metamonada, started with a mitochondrion of alphaproteobacterial origin. This finding implies that the endosymbiotic uptake could have played a role in the development of at least some common eukaryotic characteristics, along symbiogenic lines.

## Materials and methods

### Custom database

A custom database was built to cover the diversity of known organisms (Table S2) but enriched with Alphaproteobacteria to increase the sensitivity for genes affiliated with this group. Genome-derived proteomes were preferred, however, if not available, protein datasets from transcriptomes, single-cell amplified genomes and transcriptomes, or expressed sequence tags were included.

### Analyses of ancestral Metamonada proteins

OGs of metamonads (Novák et al. 2023) were filtered to contain at least one member of each of the three metamonad subclades Parabasalia, Fornicata, and Preaxostyla. These represented putative 1,399 ancestral metamonad OGs (amOGs, Table S1). Each OG was aligned with homologs from the custom database, and the alignments and preliminary phylogenetic trees were quality filtered (Figure S3). The resulting 965 OGs (Table S3b) were aligned by Muscle and trimmed by trimAl (Figure S3). Final phylogenetic analyses were inferred in IQ-TREE v2.2.0 (Nguyen et al. 2015) using the LG+C20+G4 model with 1,000 UFB replicates (Hoang et al. 2018) and at least 5,000 iterations. The affiliation of an OGs to prokaryotes (“affiliation of amOG” column in Tables S3b and S4) was assessed by objective criteria schematically described in Fig. 1.

To identify their functions, alignments of metamonad-specific OGs together with identified hits were subjected to HHblits searches (Remmert et al. 2012) in default settings.

### Analyses of MRO proteins of Metamonada

As a seed dataset for analyses focused on the MRO proteins, we used experimentally determined proteomes of Metamonada. Robust sets of MRO proteins for Parabasalia and Fornicata were prepared by overlapping the data of species within these groups (Tables S5 and S6). The published *P. pyriformis* MRO proteome (Zítek et al. 2022) represented the set for Preaxostyla MRO proteins. The final set of Metamonada MRO proteins was created by combining MRO sets of Fornicata, Parabasalia, and Preaxostyla together with additional proteins confidently predicted to be MRO-targeted in previous publications (Füssy et al. 2021; Stairs et al. 2021; Novák et al. 2023; Makki et al. 2024; Williams et al. 2024). Three proteins with complicated evolutionary histories (pyruvate:ferredoxin oxidoreductase, [FeFe] hydrogenase, and acetyl-coenzyme A synthetase) (Hug et al. 2010; Leger et al. 2017; Novák et al. 2023) were not included. This yielded 97 groups of *bona fide* MRO proteins. Each was aligned with homologs from the custom database, and the alignments and preliminary phylogenetic trees were quality filtered (Figure S3). Final alignments and trees were built in the same way as for ancestral metamonad OGs and were evaluated (“affiliation of MROG” column in Table S7b) using the same rules (Fig. 1), with the exception of criterion 1ii, which was not applied.

## Supplementary Material

msag175_Supplementary_Data

## Data Availability

The alignment and tree files were deposited in Figshare: https://figshare.com/projects/Metamonad_ancestral_OGs_and_MRO_proteins/272362. Any additional information is available from the corresponding authors.
