# Supplementary material for: Phylogenetic ancestry of Metamonada proteins points to a common origin of mitochondria in all eukaryotes
Source: Mol Biol Evol. 2026 Jul 17;43(8):msag175. doi: 10.1093/molbev/msag175 (PMC13428257; doi:10.1093/molbev/msag175)
Supplement: msag175_Supplementary_Data [file msag175_supplementary_data.zip › Fig.S1.pdf]

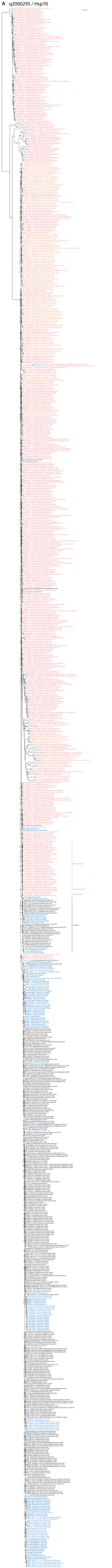

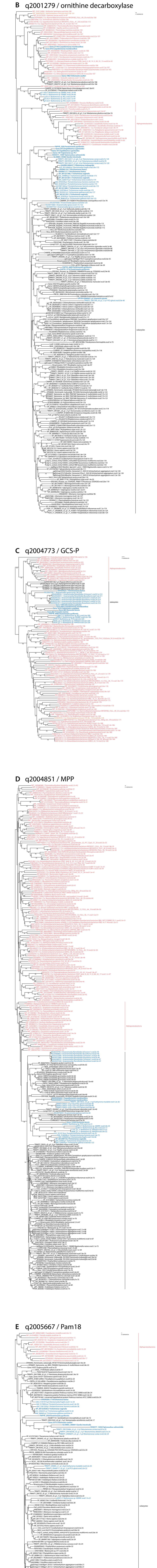

Fig. S1. Phylogenetic analyses documenting alphaproteobacterial affiliation of amOGs. Maximum-likelihood phylogenetic trees are shown with UFB supports if ≥80. Metamonads are in blue with sequences from the original OG (Novák, et al. 2023) in bold face, other eukaryotes in black, Bacteria in red, and Archaea in orange. The amOGs are indicated by the vertical lines labelling the clades.
