# Supplementary material for: Phylogenetic ancestry of Metamonada proteins points to a common origin of mitochondria in all eukaryotes
Source: Mol Biol Evol. 2026 Jul 17;43(8):msag175. doi: 10.1093/molbev/msag175 (PMC13428257; doi:10.1093/molbev/msag175)
Supplement: msag175_Supplementary_Data [file msag175_supplementary_data.zip › Fig.S3.pdf]

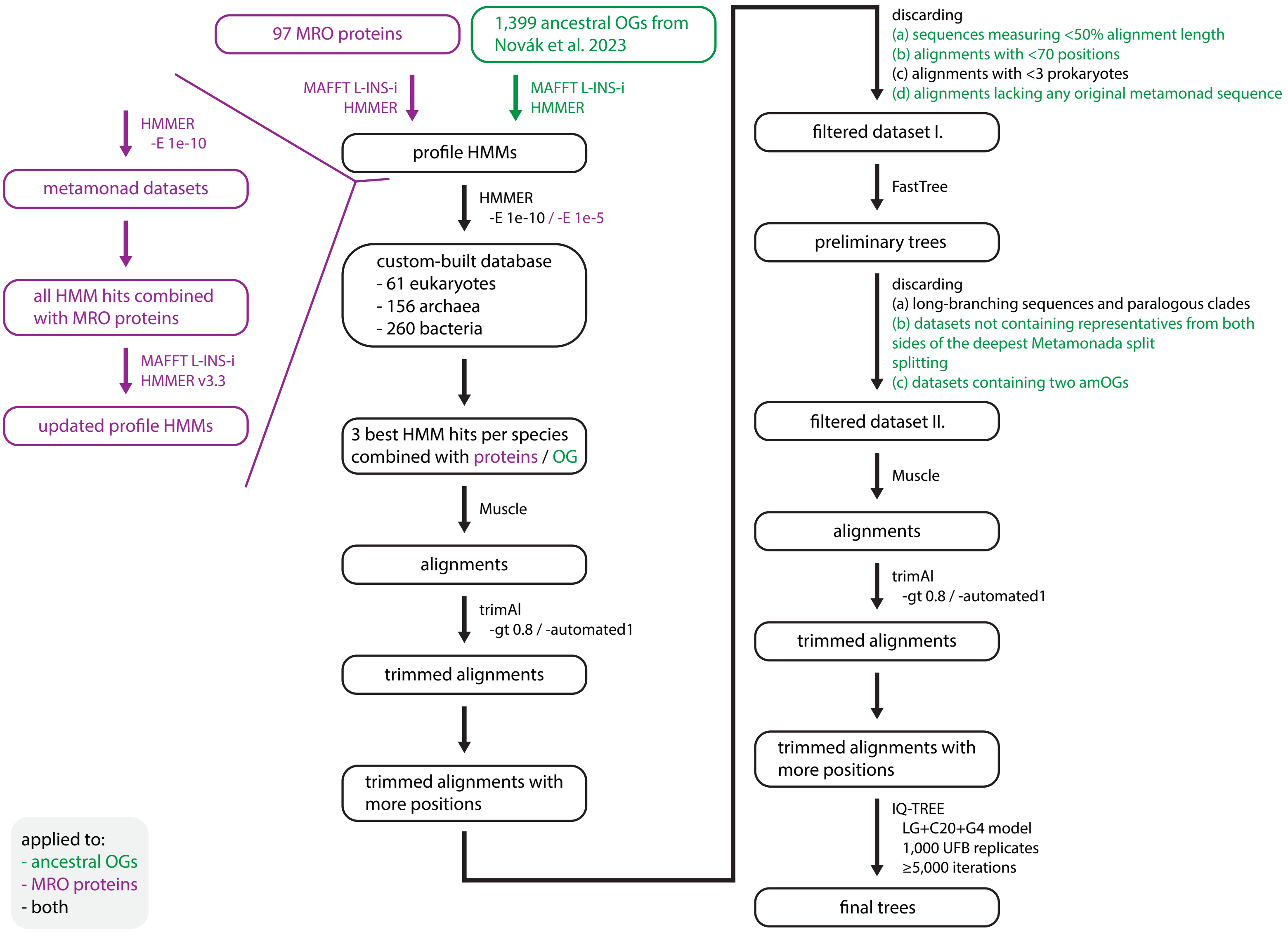

Fig. S3. Scheme of the phylogenetic pipeline. Sequences of each ancestral OG (green) or MRO proteins (purple) were aligned by MAFFT v7.110 (Katoh and Standley 2013), and a profile Hidden Markov model (HMM) was built from the alignment. Since some of the groups of MRO proteins contained very few sequences (e.g., two), one extra round of HMM searches was conducted for these MRO proteins (purple). Profile HMM was used for HMMER search (-E 1e-10) in the custom-built database. In the few cases of MRO proteins with no retrieved hits, the E-value was increased (-E 1e-5). Three best hits per organism were combined with an OG or MRO proteins and aligned by Muscle v5.1 (Edgar 2022). Alignment was trimmed by trimAl v1.2rev59 (Capella-Gutiérrez, et al. 2009) using two options (-gt 0.8 and -automated1), and the result retaining more positions was retained. Sequences measuring <50% of the alignment length were removed (a). At this step, the ancestral OG alignment was discarded if (b) contained <70 positions; or (c) contained <3 prokaryotes; or (d) lacked any "original" metamonad sequence from (Novák, et al. 2023) (**Table S3A**). The MRO protein alignment was discarded if (c) contained <3 prokaryotes (**Table S7A**). The alignments, which passed, were used for inferring preliminary phylogenetic trees using FastTree v2.1.8 (default settings). Further filtering was performed based on the tree topologies and the following changes were made: (a) long-branching sequences and paralogous clades were removed from alignments; (b) alignments of ancestral OGs without representatives from both sides of the deepest Metamonada split (i.e., Parabasalia/Anaeramoebae and Fornicata/BaSk/Preaxostyla) were removed; (c) alignments containing two paralogous clades containing ancestral metamonad groups were split into two. For MRO protein alignments, only criterion (a) was applied. The 965 ancestral OGs (**Table S3B**) and 63 MRO protein datasets (**Table S7B**), which passed this step, were aligned and trimmed as before. Final phylogenetic analyses were inferred in IQ-TREE v2.2.0 (Nguyen, et al. 2015).
