# Supplementary material for: Phylogenetic ancestry of Metamonada proteins points to a common origin of mitochondria in all eukaryotes
Source: Mol Biol Evol. 2026 Jul 17;43(8):msag175. doi: 10.1093/molbev/msag175 (PMC13428257; doi:10.1093/molbev/msag175)
Supplement: msag175_Supplementary_Data [file msag175_supplementary_data.zip › Supplementary References.docx]

References:

Aurrecoechea C et al. GiardiaDB and TrichDB: integrated genomic resources for the eukaryotic protist pathogens *Giardia lamblia* and *Trichomonas vaginalis*. *Nucleic Acids Res.*2009:37:D526-530. <https://doi.org/10.1093/nar/gkn631>.

Beltrán NC et al. Iron-Induced changes in the proteome of *Trichomonas vaginalis* hydrogenosomes. *PLoS One.*2013:8:e65148. <https://doi.org/10.1371/journal.pone.0065148>.

Capella-Gutiérrez S, Silla-Martínez JM, Gabaldón T. trimAl: a tool for automated alignment trimming in large-scale phylogenetic analyses. *Bioinformatics.*2009:25:1972-1973. <https://doi.org/10.1093/bioinformatics/btp348>.

Edgar RC. Muscle5: High-accuracy alignment ensembles enable unbiased assessments of sequence homology and phylogeny. *Nat Commun.*2022:13:6968. <https://doi.org/10.1038/s41467-022-34630-w>.

Fang YK et al. Responding to a zoonotic emergency with multi-omics research: *Pentatrichomonas hominis* hydrogenosomal protein characterization with use of RNA sequencing and proteomics. *OMICS.*2016:20:662-669. <https://doi.org/10.1089/omi.2016.0111>.

Jedelský PL et al. The minimal proteome in the reduced mitochondrion of the parasitic protist *Giardia intestinalis*. *PLoS One.*2011:6:e17285. <https://doi.org/10.1371/journal.pone.0017285>.

Katoh K, Standley DM. MAFFT multiple sequence alignment software version 7: Improvements in performance and usability. *Mol Biol Evol.*2013:30:772-780. <https://doi.org/10.1093/molbev/mst010>. Kučerová J et al. The divergent ER-mitochondria encounter structures (ERMES) are conserved in parabasalids but lost in several anaerobic lineages with hydrogenosomes. *BMC Biol.*2023:21:259. <https://doi.org/10.1186/s12915-023-01765-1>.

Kučerová J et al. Characterization of the sTim/MIA pathway in Metamonada reveals different evolutionary adaptations to anaerobiosis. *Curr Biol.*2025:35:5734-5749.e6. <https://doi.org/10.1016/j.cub.2025.10.027>.

Martincová E et al. Probing the biology of *Giardia intestinalis* mitosomes using In vivo enzymatic tagging. *Mol. Cell. Biol.*2015:35:2864-2874. <https://doi.org/10.1128/mcb.00448-15>.

Motyčková A et al. Adaptation of the late ISC pathway in the anaerobic mitochondrial organelles of *Giardia intestinalis*. *PLoS Pathog.*2023:19:e1010773. <https://doi.org/10.1371/journal.ppat.1010773>.

Rada P et al. The core components of organelle biogenesis and membrane transport in the hydrogenosomes of *Trichomonas vaginalis*. *PLoS One.*2011:6:e24428. <https://doi.org/10.1371/journal.pone.0024428>.

Schneider RE et al. The *Trichomonas vaginalis* hydrogenosome proteome is highly reduced relative to mitochondria, yet complex compared with mitosomes. *Int J Parasitol.*2011:41:1421-1434. <https://doi.org/10.1016/j.ijpara.2011.10.001>.
